# A periodic Markov model to formalize animal migration on a network

**DOI:** 10.1098/rsos.180438

**Published:** 2018-06-13

**Authors:** Andrea Kölzsch, Erik Kleyheeg, Helmut Kruckenberg, Michael Kaatz, Bernd Blasius

**Affiliations:** 1 Department of Migration and Immuno-Ecology, Max Planck Institute for Ornithology, Am Obstberg 1, 78315 Radolfzell, Germany; 2 Group of Mathematical Modelling, Institute for Chemistry and Biology of the Marine Environment, Carl von Ossietzky University Oldenburg, Carl-von-Ossietzky-Straße 9-11, 26111 Oldenburg, Germany; 3 Institute for Wetlands and Waterbird Research e.V. (IWWR), Am Steigbügel 3, 27283 Verden(Aller), Germany; 4 Vogelschutzwarte Storchenhof Loburg e.V., Chausseestraße 18, D-39279 Loburg, Germany

**Keywords:** spatial migration network, graph theory, periodic Markov process, white stork, greater white-fronted goose

## Abstract

Regular, long-distance migrations of thousands of animal species have consequences for the ecosystems that they visit, modifying trophic interactions and transporting many non-pathogenic and pathogenic organisms. The spatial structure and dynamic properties of animal migrations and population flyways largely determine those trophic and transport effects, but are yet poorly studied. As a basis, we propose a periodic Markov model on the spatial migration network of breeding, stopover and wintering sites to formally describe the process of animal migration on the population level. From seasonally changing transition rates we derived stable, seasonal densities of animals at the network nodes. We parametrized the model with high-quality GPS and satellite telemetry tracks of white storks (*Ciconia ciconia*) and greater white-fronted geese (*Anser a. albifrons*). Topological and network flow properties of the two derived networks conform to migration properties like seasonally changing connectivity and shared, directed movement. Thus, the model realistically describes the migration movement of complete populations and can become an important tool to study the effects of climate and habitat change and pathogen spread on migratory animals. Furthermore, the property of periodically changing transition rates makes it a new type of complex model and we need to understand its dynamic properties.

## 1. Introduction

Animal migration is a large-scale biological phenomenon that has fascinated humankind since ancient times. Thousands of species of birds, reptiles and mammals perform seasonal migrations, amounting to millions of individuals travelling over hundreds and often thousands of kilometres following highly predictable time schedules [1]. These mass displacements of animals have many consequences for the animals themselves, the ecosystems they visit, and also for humans [2]. While migrating, animals modify local food-web structures and predator–prey interactions (trophic effects) and transport a wide variety of non-pathogenic and pathogenic organisms, such as plant seeds and parasites (transport effects). In those respects, ‘stepping stone' migrants are especially important, because they visit and modify conditions at several stopover sites along their route where they spend days or weeks to rest and replenish their fuel reserves [3–5].

Bird migration is probably the most studied type of animal movement and much work has been devoted to understanding the physiological and ecological mechanisms of the migration process, metabolism, navigation and flight [5,6]. However, many questions remain unanswered, especially regarding the spatial structure and dynamic properties of migration and stopover usage. These properties, including route choice, number of stopovers, stopover duration and migration speed are particularly relevant in the context of the trophic and transport effects of migration [2]. They largely determine the contribution to local ecosystems processes [7], population dynamics of migrants [8] and the probability of disease transmission [9,10].

The field of animal tracking with GPS and satellite telemetry is presently growing tremendously and travels of more and more migratory species are recorded [11], improving our ability to study the temporal and spatial properties of migration. However, it is always individual animals that are tracked and usually there is large individual variation in the allocation of time and energy during their travels [12]. Thus, directly studying migration on the population or species level with tracking data is difficult. It is not straightforward how the overall migration process can be formalized with quantitative models that are able to capture its original properties with a non-infinite number of parameters.

Recently, the framework of network theory has been proposed to describe the spatio-temporal process of animal migration [13–15]. Network-based tools are already successfully used in other branches of animal ecology [16], mainly animal group behaviour [17] and population connectivity [18,19]. Furthermore, the effects of, for example, climate change on the population dynamics of migrants have been quantified with network theory methods [20–22]. However, so far, full, population-level migratory flow on a network of breeding, stopover and wintering sites (nodes) that are linked by seasonal long-distance movements (links) has rarely been modelled [23,24]. Such generalized migration models could be used to study (i) migration on the level of populations or species, (ii) the effects of climate and habitat change on migrants and (iii) disease spread phenomena.

The spread characteristics of spatial networks [25] have been extensively studied for human transportation networks like disease spread on the aviation network [26–28] or bioinvasion on the global cargo ship network [29–31]. Those networks had some very highly connected nodes and groups of tightly interconnected nodes indicating robustness to random topological changes (i.e. deletion of nodes or links) and large potential for fast spread [25]. Most studies have focused on the topological characteristics of transportation networks only, but for describing animal migration as a network, transition links need to be directed, weighted and, most importantly, include timing [14,16,22]. The latter accounts for the observation that the structure of most networks is constantly changing, with link connections evolving in time or existing only intermittently [32,33]. For animal migration networks, the explicit inclusion of timing is especially important, as migration is an inherently seasonal process.

Based on our previously published conceptual ideas [14], we present a periodic Markov model on a network that phenomenologically describes the process of animal migration in time and space. It is based on seasonally changing transition rates, reflecting migratory displacements. We provide a solution for seasonally stable densities of animals in all nodes that represent breeding, stopover and wintering sites. The model was parametrized with two example datasets: GPS and satellite telemetry tracks of white storks (*Ciconia ciconia*) and greater white-fronted geese (*Anser a. albifrons*), and seasonally changing topological and network flow properties were extracted. Node degrees, shortest path distances and motif distributions revealed that the example migration networks were highly connected and efficient for fast transport during migration, but rather stationary during breeding. Thus, our migration network model conserves important properties of the migration process, rendering it highly useful to formalize the spatio-temporal movement of complete animal populations and study the effects of habitat and climate change and disease spread.

## 2. Material and methods

### 2.1. Migration model

**Figure 1.**
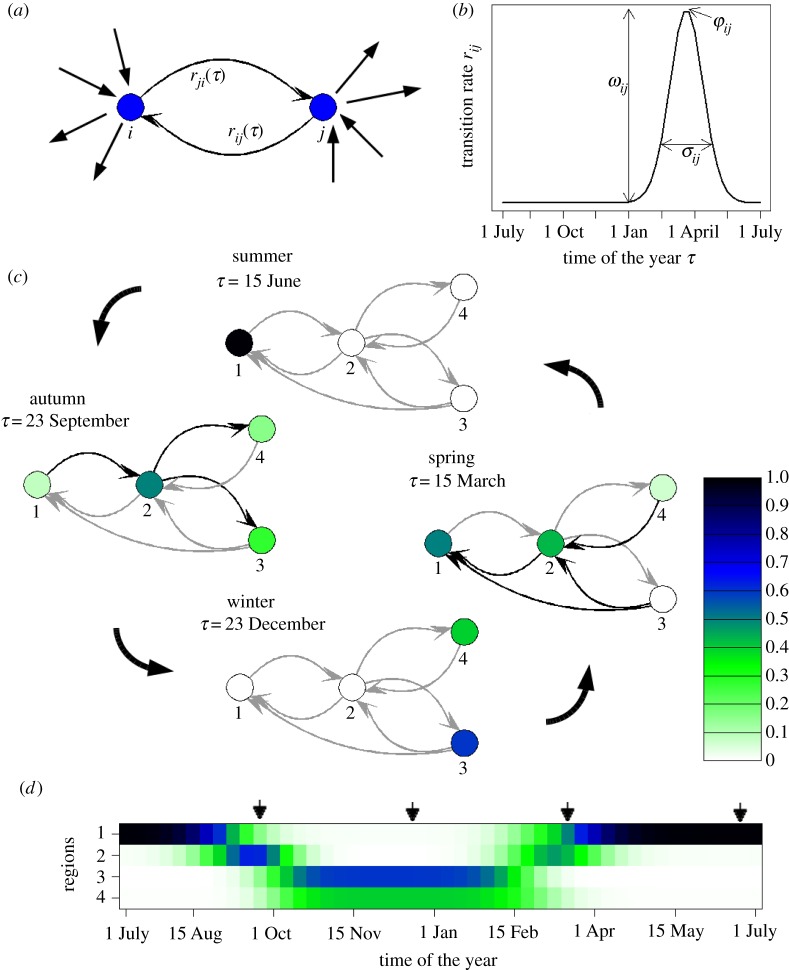
Overview of the proposed migration network model. (*a*) An isolated pair of nodes *i*, *j* that are connected by directed links weighted by the seasonally changing transition rates *r_ji_*(*τ*) and *r_ij_*(*τ*). (*b*) An example of a corresponding transition rate as function of the time of the year (season), here of a von Mises functional form (equation (2.2)). Arrows point out the three parameters that determine the function's form, namely the mean phase of the transition *φ*_*ij*_, the maximal amplitude *ω*_*ij*_ and the width *σ*_*ij*_. (*c*,*d*) Model example on a simple network of four nodes connected by seven transition links and *m* = 48 time steps of about one week. The parameters are the phases *φ*_13_ = *φ*_23_ = *φ*_24_ = 1.31 (week 10), *φ*_12_ = 1.70 (week 13), *φ*_21_ = 4.58 (week 35), *φ*_32_ = 5.11(week 39), *φ*_42_ = 5.24 (week 40), and common amplitudes *ω_ij_* = 5 and widths *σ_ij_* = 0.26. (*c*) Networks with typical density patterns of a time interval of one week for each of the four seasons. One can clearly observe breeding in node 1 in summer, spring and autumn migration via node 2 and wintering in nodes 3 and 4. The densities of birds in each node are colour coded (see legend). Links are drawn in black whenever transition probabilities are larger than 0.05 in the respective time interval, else grey. (*d*) Presentation of the year-round network flow, densities are colour coded as in *c*, each row represents density changes in the respective node over time of the year. The small arrows indicate for which time intervals network plots are shown in *c*.

We propose modelling animal migration as a non-homogeneous, periodic Markov process on a geographically embedded network. The nodes of the network are discrete areas of breeding, resting and wintering, and links represent seasonal transition events—movement between the different areas [14]. Thus, we assume there is a network of *n* nodes and a cyclic process that evolves on it (see schematic presentation of a simple version of the migration model in figure 1). The model describes the time-dependent densities *N_i_*(*t*) of animals in area *i* = 1⋯*n* at time *t*, determined by the difference of outgoing and ingoing flows *J_ji_*(*t*) and *J_ij_*(*t*) between any two connected regions *j* and *i*. One can generally formulate this space discretized process as a time continuous Markov process

2.1 $${\dot{N}}_{i}(t)=\sum_{j\neq i}r_{ij}(\tau )N_{j}(t)-\sum_{j\neq i}r_{ji}(\tau )N_{i}(t),$$

where the migration flows have been written as *J_ij_*(*t*) = *r_ij_*(*τ*) *N_j_*(*t*), with *r_ij_*(*τ*) being the transition rate from node *j* to *i* at season *τ* with τ=tmodT (figure 1*a*). That is, the transition rates *r_ij_*(*t*) = *r_ij_*(*t* + *T*) are assumed to be positive, non-homogeneous (i.e. time-dependent) and cyclic in time (period *T* = 2*π*). Time units are adapted to cyclic statistics with 2*π* corresponding to 1 year. Note that by summation over all nodes (equation (2.1)), one can show that the total animal population size remains conserved, $\sum_{i}N_{i}(t)=1.$ If the process is stationary, the densities converge into seasonal stationary states $N_{i}^{*}(\tau )$.

As a first approximation, we consider the timing of movements between sites as normally distributed, thus the transition rates *r_ij_*(*τ*) become von Mises functions (figure 1*b*)

2.2 $$r_{ij}(\tau )=\omega_{ij}\;\exp\;\left(\frac{\cos(\tau -\varphi_{ij})-1}{\sigma_{ij}^{2}}\right).$$

Here, *φ_ij_* is the main transition phase (season), *σ_ij_* characterises the length of the major transition period and *ω_ij_* is the maximum transition intensity (amplitude) that is typically realized during *τ* ≈ *φ_ij_*. This functional form of *r_ij_*(*τ*) also describes the extreme cases of the *δ*-function (*σ_ij_* → 0), when all animal movements on a given connection occur at the same time instance, and the uniform distribution (*σ_ij_* → ∞), when the transition rates are time-independent.

### 2.2. Numerical implementation as a time-discrete process

To implement this process numerically, we formulate a time-discrete version of the continuous process (equation (2.1)). For this we split each year into *m* smaller time intervals of equal length Δ*τ* (*T* = *m* · Δ*τ*). The density *N_i_*(*t* + Δ*τ*) of animals at site *i* in the next time interval is then given as the sum of the animals that migrate into the site plus the animals that stay in the site (and do not move in this time interval). This can be derived from equation (2.1) into a Markov chain equation [34]

2.3*a* $$N_{i}(t+\Delta \tau )-N_{i}(t)=\sum_{j\neq i}p_{ij}(\tau ;\Delta \tau )N_{j}(t)-\sum_{j\neq i}p_{ji}(\tau ;\Delta \tau )N_{i}(t)$$

or

2.3*b* $$N_{i}(t+\Delta \tau )=\sum_{j\neq i}p_{ij}(\tau ;\Delta \tau )N_{j}(t)+p_{ii}(\tau ;\Delta \tau )N_{i}(t),$$

where *p_ij_*(*τ*;Δ*τ*) denotes the transition probability to make a movement from node *j* to *i* at season *τ* within the finite time interval of length Δ*τ* and $\sum_{j\neq i}p_{ij}(\tau ;\Delta \tau )+p_{ii}(\tau ;\Delta \tau )=1$. The transition probabilities *p_ij_*(*τ*;Δ*τ*) of the time-discrete process (equation (2.3*a*)) are related to the transition rates *r_ij_*(*τ*) of the time continuous process (equation (2.1)) by an exponential transformation [34]

2.4 $$P(\tau ;\Delta \tau )={\text{e}}^{R(\tau )\cdot \Delta \tau }.$$

Here, *P*(*τ*;Δ*τ*) = (*p_ij_*)*_ij_* represents the *n* × *n* matrix of transition probabilities and *R*(*τ*) = (*r_ij_*)*_ij_* the corresponding *n* × *n* matrix of transition rates, with diagonal elements defined as $r_{ii}=-\sum_{j\neq i}r_{ij}(\tau )$. Because of the monotone transformation by an exponential (equation (2.4)), the transition probabilities *p_ij_*(*τ*;Δ*τ*) are naturally related to the von Mises function (equation (2.2)).

To solve for the stationary distribution of densities in all sites, we plug the (empirically determined) transition rates (equation (2.2)) into equation (2.4) and determine the instantaneous transition probability for each discrete time interval, giving rise to a set of *m* probability matrices *P*(*τ*;Δ*τ*). Next, we calculate the Poincaré map *S*(*τ*) from one year to the next at each time step *τ* by matrix multiplication of the *m* subsequent transition matrices

2.5 $$S(\tau )=\prod_{k=0}^{m-1}P(\tau +k\Delta \tau ;\Delta \tau ).$$

Each of these resulting *m* matrices *S*(*τ*) is a regular stochastic matrix and by the Perron–Frobenius theorem has a dominant eigenvalue *λ*(*τ*) = 1 [35]. Then, the seasonal stationary distribution of densities $N_{i}^{*}(\tau )$ in sites *i* at season *τ* is obtained as the corresponding right eigenvector of *S*(*τ*),

2.6 $$N^{*}(\tau )=S(\tau )N^{*}(\tau ).$$

### 2.3. Simple model example

How our model formalizes the population migration process and how seasonal stable densities are driven by network topology and the transition parameters $\varphi_{ij}$, *σ_ij_* and *ω_ij_* can be followed in a simple example (figure 1*c*,*d*). There are four sites (nodes) that are connected by von Mises seasonal transition rates (see figure 1 for parameters) with Δ*τ* being about one week (*m* = 48). In the summer months, all animals are in node 1. During autumn migration they move via the stopover site, node 2, to the wintering sites, nodes 3 and 4. Because the transition from node 2 to 3 starts somewhat earlier than from node 2 to 4 (*φ*_32 _< *φ*_42_) winter densities are higher in node 3. During spring migration, animals from node 4 have to migrate via the stopover site, node 2, to the summer region, node 1, whereas animals from node 3 can move via node 2 or directly to node 1. Therefore, node 3 empties earlier in spring than node 4 and the stopover site, node 2, is less used than in autumn. This example shows how we were able to quantify the complex spatio-temporal flow of migratory animals on a seasonal network with only seven model parameters.

### 2.4. Example migration tracks

To examine our model's ability to portray real stepping stone migration patterns [5], we used a set of quality filtered [36] satellite telemetry and GPS tracks of white storks and greater white-fronted geese. Both are large, long-distance migrants that use several stopover sites along their migration route [37]. We selected full-year trajectories of adult birds because our model requires data that are evenly distributed throughout a full calendar year. For the white stork this yielded 32 yearly tracks of 12 adult individuals [38] and for the white-fronted goose data encompassed 8 yearly tracks of seven adult individuals [39].

### 2.5. Determination of network nodes

**Figure 2.**
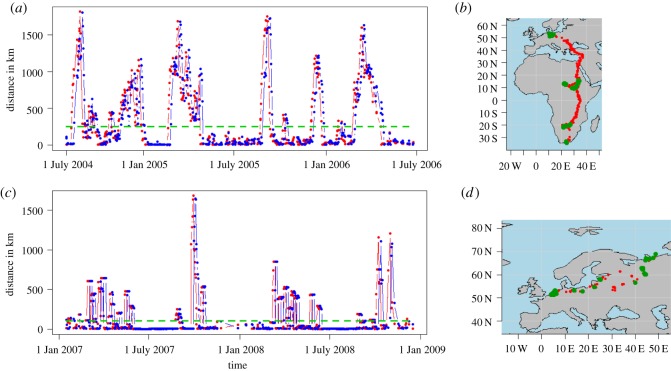
Flight velocities and derived resting locations. (*a,c*) Time series of daily flight distances of movement towards (red) and from (blue) the best daily bird position at each day, for a selected 2-year track of a white stork (*a*) and a greater white-fronted goose individual (*c*). Positions were selected as resting locations when the approaching and departing velocities were smaller than a threshold (horizontal green line) of 50 km d^−1^ for the stork and 20 km d^−1^ for the goose. On the maps aside (*b,d*) the selected breeding, resting and wintering locations are mapped as green dots (red dots depict positions of fast migratory movement) for the individual stork (*b*) and goose (*d*).

For both datasets, the breeding, wintering and resting areas, i.e. the nodes of the network, had to be determined. This could generally be done using observations or environmental data, but for simplicity, we here used an approach based solely on the migration trajectories. For each track, we first selected one best daily position (based on satellite telemetry location classes and GPS horizontal accuracy, respectively) of the bird at the end of each day and then approximated flight velocities towards the previous and next day's positions (figure 2). The flight velocities showed a distinct bimodal behaviour, of either fast migratory movement or resting, allowing the determination of resting positions based on a simple threshold value.

Best daily positions for which both velocities were less than 50 km d^−1^ for the white storks [40] and 20 km d^−1^ for the white-fronted geese [41] were selected as resting localization (see in relation to data in figure 2). Those positions were then hierarchically clustered by spatial distance and grouped at distances less than 750 km, which was the average distance between migration stopover sites. To account for outliers in our dataset, only clusters that contained more than three resting positions were selected as typical breeding, resting and wintering regions of the considered species. The selected parameters and thresholds determine the resulting network topology and should, therefore, be carefully selected based on data quality, the research question and previous knowledge of the species and their migration behaviour. Per species, we designed a time-cumulative network, only containing the links between those pairs of nodes between which at least one bird had moved, i.e. using the two sites successively.

### 2.6. Determination of seasonal transition rates

Once the network nodes and links were established, we determined the time-continuous transition rates along these connections. For this, we divided the year into *m* discrete time intervals of equal length Δ*τ* that are sufficiently small so that the transition rates can be assumed to remain constant within each interval. Because of the limited time resolution of the datasets, we selected Δ*τ* to be about one week (Δ*τ* = 0.13 if 1 year = 2*π*), giving rise to *m* = 48 seasonal time intervals. For all seasonal time intervals, we counted the numbers of animals in each of the above defined resting regions, yielding the empirical, seasonal bird densities *N_j_*(*τ*) in the network nodes. In a similar way, we counted the seasonal transition flows *J_ij_*(*τ*, *τ* + Δ*τ*) between all connected pairs of nodes for all time intervals. Based on the obtained numbers, we approximated the seasonal transition rates for each discrete time interval as

2.7 $${\tilde{r}}_{ij}(\tau )=\frac{J_{ij}(\tau ,\tau +\Delta \tau )}{N_{j}(\tau )\cdot \Delta \tau }.$$

Next, we applied circular statistics [42] to parametrize a von Mises function (equation (2.2)) specific to each link of the cumulative network with the empirically determined seasonal functions ${\tilde{r}}_{ij}(\tau )$. Thereby, we characterized the seasonal transition along each connection in terms of three numbers: the circular mean of the transition times *φ_ij_*, the circular standard deviation of the transition times *σ_ij_* and the maximum transition intensity *ω_ij_* (see electronic supplementary material, for equations). Thus, we obtained a smooth model transition rate *r_ij_*(*τ*) for any season *τ* (equation (2.2)). Finally, using the migration model (equations (2.4)–(2.6)), we obtained seasonal stationary bird densities $N_{i}^{*}(\tau )$ for each time interval *τ* and node *i* of the two migration networks. We provide R code for determination of the migration network model in the electronic supplementary material.

### 2.7. Characterization of migration network models

To evaluate whether our parametrized models conform to the main characteristic of migration systems, namely shared, directed seasonal movement between breeding and wintering regions, we assessed the estimated population densities and a selection of network measures for the two cumulative networks as well as the two sets of seasonal time-dependent networks. Season-specific migration network topologies were derived such that a pair of nodes was connected by a directed link from *j* to *i* whenever *p_ij_*(*τ*;Δ*t*) > 0.05. Those were then merged into time-cumulative networks by overlap.

Of several standard network measures that can help to further characterize migration networks [16], we here focus on one property of the cumulative network that can easily be calculated, the shortest directed topological path distance. This is the minimum number of links (and stopover sites) that a migrating animal has to traverse to move between two nodes. Comparatively short paths between breeding and wintering regions indicate migration. Important network measures characterizing high possible movement between nodes are node degree (number of links that start or end in a node) and the size of the giant component (size of the largest connected sub-network). For our purposes, the seasonal changes of those measures are most indicative of migration, namely times of high migration activity versus times of breeding and winter, thus we determined mean degree and giant component size of the season-specific networks.

For further confirmation that our networks allow quick directed migration flow, we determined their motif distributions [43], i.e. the distribution of differently connected triplets. If such three-node sub-networks are, for example, connected by two directed links to allow flow through the middle node, this is an indication of a transportation network, as our migration networks should be.

We determined each node's degree and betweenness centrality, which is defined as the number of topologically shortest paths in the network that pass the respective node [44]. This measure characterizes how much each node contributed to network connectivity, flow and clustering. Further, we determined the cumulative density and the staying time after the highest densities, which indicate the nodes that were used often and by a large number of birds. For more detailed flow characterization, we determined the first passage times [35] between two different nodes, as well as return times to the same node. As those values varied with starting season *τ*, we selected the first passage and return times starting in the season of highest $N_{i}^{*}(\tau )$ to provide minimum values.

### 2.8. Model validation and sensitivity

To validate the model's appropriateness for mapping animal migration, we explored how well empirical and fitted non-zero transition rates ${\tilde{r}}_{ij}(\tau )$ and *r_ij_*(*τ*) matched using paired Wilcoxon's tests. Comparisons of the distribution of the number of birds in the data with the modelled, stable seasonal densities $N_{i}^{*}(\tau )$ were performed using *G*-tests [45].

Sensitivity of model outcomes $N_{i}^{*}(\tau )$ to changes of the phases *φ_ij_*, widths *σ_ij_* and amplitudes *ω_ij_* of single transition rates was determined as follows. For single transition rates, in turn, one of the three parameters was varied and model outcomes were compared with the original ones by *G*-tests. Transition rate amplitudes were modified multiplicatively by values from 0.01 to 100, phases were shifted additively by values out of [ − 1.58, + 1.58] (±3 months) and transition rate widths were increased or decreased by values of the same interval (we forced a truncation at a minimum value of 1 day = 0.017). The outcomes were considered sufficiently close to the original parametrizations' results if more than 67% of the *G*-tests for the distributions in each season *τ* had a *p* > 0.1.

## 3. Results

### 3.1. Migration network models

**Figure 3.**
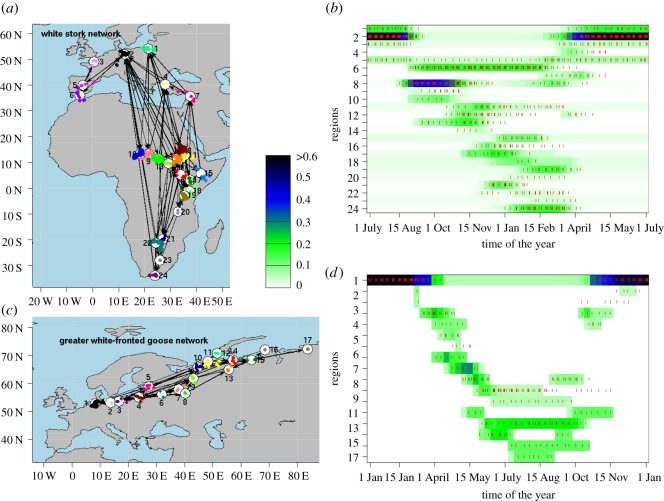
Migration networks and stable seasonal population densities. (*a*,*c*) Cumulative migration network with resting location clusters (different colour per site) and network nodes for (*a*) the white stork and (*c*) the greater white-fronted goose dataset. Arrows indicate the links of the cumulative network. (*b*,*d*) Flows from the seasonal migration model on the network of the white stork (*b*) and the greater white-fronted goose (*d*) are indicated by bird densities in each node for a number of *m* = 48 time intervals during the year. The small red dots indicate days where birds were observed in the respective sites from the tracking data. Note that the density colour bar has been rescaled to maximum local density of 0.6.

The determined cumulative migration networks (figure 3*a*,*c*) of breeding, resting and wintering regions showed much individual variability for both species. The network of stork migration consisted of *n = *24 nodes and *l = *96 directed links and the goose network had *n *= 17 nodes and *l *= 53 links, whereas individuals of both species used only up to nine sites per year.

**Figure 4.**
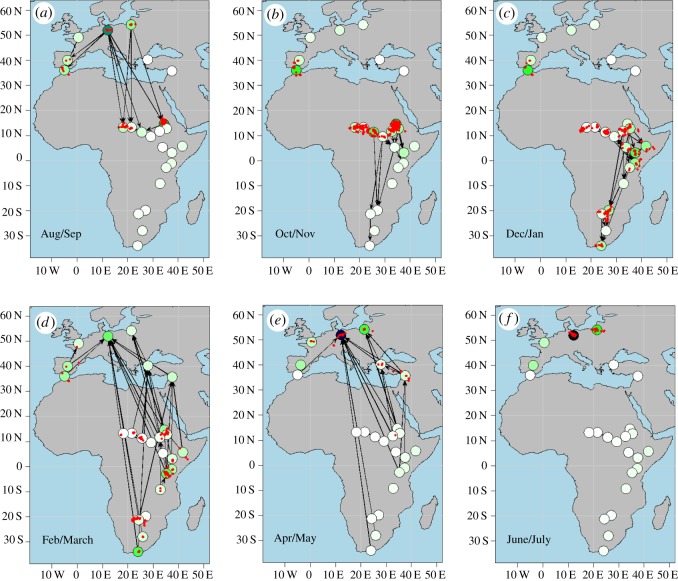
(*a*–*f*) Season-specific, two-month cumulated networks of the white stork. Colouring of the nodes indicates modelled, stable seasonal densities of birds there (see label in figure 3, right panel). A link is drawn here whenever modelled transition probabilities were larger than 0.05 for any one-week period within the considered time interval. The change in the connectivity thus indicates different migration phases. Red dots show real data positions of white storks within the respective time period.

The seasonal flow of birds in the networks (figure 3*b*,*d*) indicated cyclic movement in time of all individuals. At some times of the year, all birds remained in a few nodes only, whereas during migration much movement happened. Furthermore, the flow of birds was not just linear along a number of nodes, but a lot of branching occurred, especially in the stork network. Some of the nodes were frequented by a large number of birds and for a long time, but others seem to have simply been short-time resting areas. Times when birds actually used a node seemed to overlap well with model density estimations (small red lines in figure 3*c*,*d*). Six season-specific, cumulated sub-networks of the stork model (figure 4) outline how the model generally maps migratory flow characteristics.

### 3.2. Characterization of migration network models

**Figure 5.**
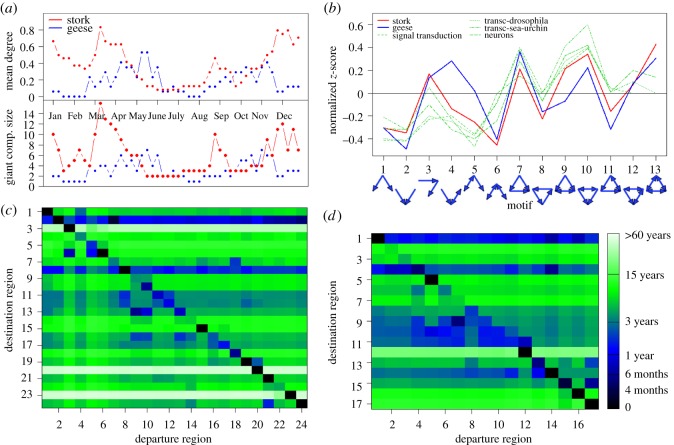
Characteristics of the white stork and greater white-fronted goose migration network models. (*a*) Mean degree and giant component size of the season-specific networks for storks (red) and geese (blue). Note how they change with times of migration, breeding and wintering. (*b*) Motif distributions of the two cumulative migration networks which are similar to those of other networks of real transportation processes. (*c*,*d*) First passage times, each starting from the season of highest density in the outgoing node that characterize the migration process of the storks (*c*) and geese (*d*). They indicate from which regions transition times were long (blue-black) or shorter (green-white). Diagonals indicate return times after departure at the season of highest density.

In comparison to the sizes of the networks, the mean shortest (directed) path lengths were very small (2.47 for storks, 2.46 for geese). When looking at shortest paths between the breeding and wintering sites, first migration properties of our networks appeared: between nodes 2 (Middle European breeding region) and 8 (Sudan wintering and staging area) of the stork network only one link had to be crossed in either direction, indicating direct, possibly fast migration. Movement between node 2 and the southernmost node 24 (South Africa) could be done through three links in autumn, but by one direct link in fast spring migration. Similarly, in the goose network, the shorted path between node 1 (Middle European wintering region) and node 11 (breeding region) had length 3 in spring (many stopovers), but only length 1 in autumn (quick directed back migration). Mean degree and the size of the giant component of the time changing networks (figure 5*a*) further illustrated times of migration movement and when birds were moving slowly.

Betweenness centrality (electronic supplementary material, table S1) revealed that for the stork network, nodes 2 (Middle European breeding region) and 8 (Sudan wintering and staging area) were especially important, whereas nodes 3 (French breeding area) and 20 (staging area close to Lake Malawi) were not central at all. For the goose network, nodes 1 and 4 were of high importance and nodes 2 and 12 not. Cumulative densities (in units of density × time intervals; range = [0; 48], 48 indicating that all individuals were in the same node all year) in highly central nodes were mostly large (12.66 for stork node 2, 12.66 for goose node 1; note that the equality of the two values is coincidental), but staying times were more intermediate (2.23 = 130 days for stork node 2, 2.50 = 145 days for goose node 1). Longer staying times were mostly obtained for less connected nodes, as calculated densities were stuck there due to small transition rates obtained from only one bird in our dataset taking that route.

The motif distributions confirm that there are a large number of sub-graphs that represent transit movement (sub-networks 3, 4), which is an important property of migration. Additionally, patterns of branching character (sub-graphs 7, 9 and 10) are overrepresented in comparison to random graphs of similar structure. It can be observed that the motif distribution (figure 5*b*) falls into the second superfamily [46] which also includes the signal-transduction and neurons networks of simpler organisms.

First passage times after maximum starting density (figure 5*c*,*d*) indicated which of the nodes were reached on average more frequently and quicker than others. They were again nodes 2 and 8 for the stork network and nodes 1 and 4 for the geese. Return times (diagonals in figure 5*c*,*d*) were generally shorter than first passage times, indicating possible backwards movements due to long transition rate distributions (i.e. large *σ_ij_*).

### 3.3. Model verification and sensitivity analysis

The counted and the fitted transition rates ${\tilde{r}}_{ij}(\tau )$ and *r_ij_*(*τ*) were very similar (Wilcoxon tests: *W*_s_ = 9040, *p*_s_ = 0.23; *W*_g_ = 1870, *p*_g_ = 0.29) with average deviations of only 10% (± 14%) for the stork network and 7% (±13%) for the goose network. Also resulting estimated densities $N_{i}^{*}(\tau )$ were very similar to the numbers of counted birds in each region at the respective seasons (*G*-tests: ${\left\langle G\right\rangle }_{s}=15.77$ (*p* = 0.76) and ${\left\langle G\right\rangle }_{g}=11.01$ (*p* = 0.96); figure 3*b*,*d*), with average differences of 0.65 (storks) and 0.67 (geese) individuals (of *N* = 32 and *N* = 8, respectively). Tests of 87.5% and 100% of the seasonal stork and goose models versus densities, respectively, had *p* > 0.1.

Both migration networks were robust to single changes in the parametrizations of the phases *φ_ij_* and amplitudes *ω_ij_* of the transition rates, with few exceptions only. For the stork network, only a decrease of *φ*_52_ by more than 0.48 (= 28 days), an increase of *ω*_62_ (by more than *25) or a decrease of *ω*_26_ (by more than *0.03) led to significant model alterations. For the goose network, all phases could be decreased or increased up to three months without significant change in $N_{i}^{*}(\tau )$, but decreased amplitudes *ω*_16_ and *ω*_15,17_ (both by more than *0.03) altered model outcomes significantly.

On the contrary, model outcomes were relatively sensitive to changes in transition rate widths *σ_ij_*. The subtraction of small values from single *σ_ij_* often led to *σ_ij_* ≈ 0, which is one possible cause of its greater influence on model behaviour. For the stork network, addition to *σ*_52_ (+0.241 = 14 days) changed $N_{i}^{*}(\tau )$ notably, as well as addition to *σ*_81_ (+1.102 = 64 days) and *σ*_17,15_ (+1.067 = 62 days) for the goose network. Subtraction of very small values (2–14 days) to most *σ_ij_* had great impact on model outcome. However, a few *σ_ij_* were more robust to changes, e.g. *σ*_8,12_, *σ*_13,9_ and *σ*_10,9_ for the stork network and *σ*_13_ for the goose network (from −0.70 to −1.03 = 40–60 days). These sensitivities indicate that the ranges of inter-node transition times strongly affect migrant seasonal abundance, in the model as well as in migration itself.

## 4. Discussion

Combining methods from non-homogeneous Markov modelling and network theory, we have developed a spatio-temporal periodic animal migration flow model. From seasonal transition rates between breeding, stopover and wintering sites, we derived stable seasonal densities of animals, thus generalizing population migration flow. The model was parametrized with high-quality, year-round tracking data of two species of long-distance migrants. Cumulative network characteristics of the two resulting networks and flow processes are very similar and show properties of a seasonal migration system. Thus, our model phenomenologically maps migration movement in a realistic way and can be used for further applications to explore the effect of migration on traversed ecosystems and vice versa.

In comparison to already existing migration network models, we have solved one of their main limitations, namely the use of suitable empirical data (i.e. tracking data) for migratory connectivity and movement quantification [20], rather than expert knowledge [21] or ringing and count data [23]. Thus, also assumptions about migration flow characteristics like optimal flow or the general flux law based on habitat attractiveness [21,22] are not necessary. Other than previous systematic studies on network patterns and effects of topological changes [15,22], our networks are very close to the available datasets and allow for predictions of realistic change effects or spread characteristics in the modelled systems.

Different from the existing explicit population network models [20–23], our model is a pure movement model that obeys the conservation law, i.e. we neglect population growth and consider total population size constant during the timescale of the analysis. For our datasets of adult storks and geese, this assumption is sensible in relatively stable environments, as those species are long-lived. However, if individuals are short-lived and population growth is strong and not similar for individuals using different migration routes or timing, resulting movement patterns might be unrealistic in the long run. If comparing model outcomes of different, shorter time periods, one might be able to detect such changes in long-term migratory dynamics like population size, stopover usage or timing.

Further assumptions of our model are the Markov property, which implies that birds use no memory of their previous whereabouts but only season and their present state for the decision regarding where to go next or if to stay, and the independence of the transition rates from the present densities in the nodes, thus we disregard any swarming or density dependence effects. Finally, the decision for migratory departure from breeding, stopover and wintering sites has been proposed to depend on environmental characteristics like the photoperiod and most importantly food availability and uptake [3,47], that might vary between years. Such habitat quality dependence is not explicitly accounted for in our model, but is implicitly included in the time dependence of the transition probabilities.

All assumptions were made to introduce our basic, phenomenological network model of stable migration movement. It can be readily extended with additional terms of past densities, immigration, mortality, density dependence or environmental factors. For example, an additional sink/source node can be added for integration of vital rates. However, already without any additions, the novel issue that network structure changes periodically with time makes processes on our interchanging network model highly complex.

By translating bird migration into a temporal network, we have gained access to the huge arsenal of technical and statistical tools developed by network theory. We showed that application of network indices indeed allows one to obtain useful insights into the spatio-temporal flow of birds during their migration and into the small- and large-scale organization, connectivity and its temporal dependence, which would not be straightforward to obtain with other methods. Namely, the network characteristics of our cumulative stork and goose migration models were very similar to other transportation networks, especially the aviation and cargo ship networks. Like those, our migration networks are inherently non-planar, show properties of transit and have small shortest paths. However, differently from human transportation networks, our described migration flow is strictly seasonal and there are characteristic pauses during breeding, winter and in stopovers regions that differ strongly by season and species. Surprisingly, for storks it is autumn migration, but for geese spring migration that is interspersed with several stopovers and makes the movement flow irregular rather than uniform, likely driven by environmental conditions [41].

Because GPS tracks of migratory animals become more and more ubiquitous and of higher quality [11], it will become possible to parametrize our model in more detail and possibly for different, interacting populations or species. This will allow more accurate network characterization and the evaluation of processes on this network, like cross-continental interspecific disease spread. Moreover, there are other methods to determine network nodes, i.e. breeding, stopover and wintering sites from movement trajectories [48], and it should be decided by species and population which one is most applicable. Our analyses of model sensitivity to changes in the transition rates on the network links revealed that the duration of possible transition between sites strongly affected network connectivity, whereas peak transition time and magnitude could be varied without great influence. This indicates that migrants with high plasticity in migration timing are more robust to changes.

Specific future applications of our model are plentiful, particularly in the context of trophic and transport effects of migration. By quantifying the number of individuals present at stopover sites during different seasons, one can assess the potential benefits and costs of migrating populations on local animal and plant communities. For white-fronted and other goose species this is particularly helpful in quantifying the pressure on arable fields, as well as the impact of population management at multiple spatial scales [49]. For white storks, it can help to understand where and when populations are most vulnerable to mortality and thus where and when management measures should be installed [50]. These examples can be extended to a wide range of other migrants. Obvious next steps are the combination with epidemiological models to simulate the spread of zoonotic diseases like avian influenza or West Nile virus [51], or to simulate the resilience of migratory animal populations to changes in habitat suitability at important stopover sites resulting from anthropogenic or climate-driven land-use changes [52].

In conclusion, our model provides an empirically informed quantitative tool to formalize sets of individual migration tracks into spatio-temporal dynamics of migratory populations. The periodically changing network process is a new type of complex model and we invite further theoretical studies to examine and understand its dynamic properties. We propose using our cyclic description of migration patterns in combination with the large suite of mechanistic migration models already available [53] to study different aspects of animal migration. By using recently available larger datasets and possibly more migratory species in combination, our model will show its full potential to gain important new insights into a range of current and pressing topics in the field of animal migration.

## Supplementary Material

Transition formulae, Table S1 and R Code

## Data accessibility

The GPS and satellite telemetry tracks used in the study have been uploaded to Movebank (www.movebank.org), available in the studies ‘White stork full year tracks 1994–2006 MPIO' and ‘White-fronted goose full year tracks 2006–2010 Alterra IWWR', and published in the Movebank Data Repository (doi:10.5441/001/1.k29d81dh) [38] and (doi:10.5441/001/1.kk38017f) [39].
