# Supplementary material for: A periodic Markov model to formalize animal migration on a network
Source: R Soc Open Sci. 2018 Jun 13;5(6):180438. doi: 10.1098/rsos.180438 (PMC6030295; doi:10.1098/rsos.180438)
Supplement: Transition formulae, Table S1 and R Code [file rsos180438supp1.pdf]

Electronic Supplementary Material

to

A periodic Markov model to formalise animal migration on a network

Andrea Kölzsch, Erik Kleyheeg, Helmut Kruckenberg, Michael Kaatz and Bernd Blasius

Royal Society Open Science, 2018

Transition rate estimates from circular statistics

To parameterise the network model, for each nonnegative transition link ($\exists\tau : r_{ij}(\tau) > 0$) the von Mises parameters φ_{ij}, ω_{ij} and σ_{ij} were estimated using circular statistics as follows:

$$\varphi_{ij} = \arctan\left(\frac{\frac{1}{\sum_\tau \tilde{r}_{ij}(\tau)} \sum_\tau \left(\tilde{r}_{ij}(\tau)\sin\tau\right)}{\frac{1}{\sum_\tau \tilde{r}_{ij}(\tau)} \sum_\tau \left(\tilde{r}_{ij}(\tau)\cos\tau\right)} \right) \tag{S1}$$

$$\sigma_{ij} = \sqrt{\frac{1}{\sum \tilde{r}_{ij}(\tau)} \sum_\tau \tilde{r}_{ij}(\tau)\left(\left|\tau - \varphi_{ij}\right| \bmod(2\pi)\right)^2} \tag{S2}$$

$$\omega_{ij} = \max_\tau\left(\tilde{r}_{ij}(\tau)\right). \tag{S3}$$

Here φ_{ij} is the by the transition rates weighted circular mean of the transition times from *j* to *i*, σ_{ij} the similarly weighted circular standard deviation of the transition times, and ω_{ij} is the maximum transition intensity (see also Fig. 1 in main text). Note that $\tilde{r}_{ij}(\tau)$ are empirical transition rates estimated from data.

Table S1. Centrality characteristics and staying times for each specified migration network node. Per node of the stork network and goose network we present values of betweenness centrality *bc* and degree *k*. Furthermore, the cumulative density $\sum_{\tau} N(\tau)$ indicates how much each node is frequented during the year, and staying times starting at the season of maximum density in the node (they vary with starting time) $t_{stay,\, max\, x}$ reveal how long a theoretical bird would stay in the node. These measures indicate each node's importance for connectedness, connectivity and network flow and dynamics. Values highlighted in bold mark nodes of high connectedness and importance, whereas italic numbers indicate the opposite.

**nodes**	***Network characteristics***			
**stork network**	***bc***	***k***	$\sum_{\tau} N(\tau)$	$t_{stay,\, max\, x}$
**1**	16.81	3.5	**3.74**	0.92
**2**	**231.69**	**9.0**	**12.66**	1.17
**3**	*0.00*	*1.0*	1.49	4.09
**4**	13.56	4.5	0.42	0.13
**5**	22.00	2.5	2.65	2.39
**6**	22.00	2.5	**3.87**	1.91
**7**	13.02	3.5	0.61	0.12
**8**	**121.84**	**7.5**	**4.78**	0.79
**9**	23.90	4.5	*0.28*	0.08
**10**	9.90	3.5	0.97	0.33
**11**	10.89	4.5	1.66	0.34
**12**	46.21	**6.5**	0.91	0.21
**13**	**78.00**	**6.0**	1.40	0.27
**14**	30.54	4.5	0.38	0.14
**15**	8.82	2.5	1.63	1.71
**16**	23.10	5.0	0.51	0.19
**17**	27.89	5.0	2.00	0.62
**18**	10.18	2.5	1.80	1.81
**19**	34.73	4.0	1.72	0.87
**20**	*0.96*	*2.0*	0.63	3.26
**21**	38.62	4.5	0.80	0.41
**22**	12.57	3.0	0.56	0.76
**23**	*5.67*	*1.5*	0.64	3.13
**24**	10.10	2.5	1.88	0.61
**goose network**				
**1**	**73.84**	**6.5**	**12.66**	1.31
**2**	*0.00*	2.5	1.47	1.98

**3**	10.08	3.5	0.89	1.19
**4**	**63.84**	**6.0**	2.48	0.69
**5**	17.93	3.0	1.87	1.73
**6**	2.07	2.0	1.69	1.56
**7**	6.49	2.5	0.42	0.41
**8**	12.95	2.0	2.80	1.52
**9**	30.22	3.5	**3.20**	1.27
**10**	47.50	5.5	2.22	0.55
**11**	26.86	4.0	1.67	0.57
**12**	*0.00*	1.0	1.46	3.78
**13**	23.99	2.0	2.58	1.43
**14**	19.13	3.0	**4.03**	1.29
**15**	**51.50**	4.0	2.75	0.69
**16**	10.60	1.0	**3.11**	**4.05**
**17**	*0.00*	1.0	2.43	**3.13**

R code for calculation of migration network process from daily resting locations

```
## Calculate and solve migration network from daily resting positions
## ESM of Royal Society Open Science paper
## AK 15 March 2018

## subfunctions
rast2NW <- function(daten,hcldist=750,minpos=3)
{
  require(geosphere)
  datetime <- as.POSIXct(daten$time)
  ranz <- length(daten[,1])
  entf <- tdi <- matrix(rep(0,ranz*ranz),nc=ranz)

  for (i in 1:(ranz-1))
  {
    for (j in (i+1):ranz)
    {
      entf[i,j] <-
distVincentyEllipsoid(c(daten$lon[i],daten$lat[i]),c(daten$lon[j],daten$lat
[j]))
      entf[j,i] <- entf[i,j]

      tdi[i,j] <-
abs(as.numeric(difftime(datetime[i],datetime[j],units="days")))
      tdi[j,i] <- tdi[i,j]
    }
  }
  tdik <- tdi %% 365

  #cld <- hclust(as.dist(entf/1000+tdik/10))
  cld <- hclust(as.dist(entf/1000))
  plot(cld)
  abline(h=hcldist,col=2)
  clurastdata<-data.frame(daten,"clust"=cutree(cld,h=hcldist))

  out <- which(clurastdata$clust %in%
as.numeric(names(table(clurastdata$clust))[which(table(clurastdata$clust)<m
inpos)]))
  if (length(out)>0) clurastdataS <- clurastdata[-out,] else clurastdataS
<- clurastdata
```

```
  u_nw <- unique(clurastdataS$clust)
  for (i in seq(along=clurastdataS$clust)) clurastdataS$clust[i] <-
  which(u_nw == clurastdataS$clust[i])

  nn <- unique(clurastdataS$clust)
  nw_nodes <-
data.frame("node"=1:length(nn),"midlon"=numeric(length(nn)),"midlat"=numeri
c(length(nn)))
  for (i in 1:length(nn))
  {
    nw_nodes$midlon[i] <- mean(clurastdataS$lon[clurastdataS$clust==i])
    nw_nodes$midlat[i] <- mean(clurastdataS$lat[clurastdataS$clust==i])
  }
  if (any(is.na(nw_nodes$midlat))) nw_nodes <- nw_nodes[-
which(is.na(nw_nodes$midlat)),]

  return(list("cludata"=clurastdataS,"nodes"=nw_nodes))
}

##
count_pit_rijt <- function(cludata,dtN=48)
{
  n <- length(unique(cludata$clust))
  nodes <- data.frame("clu"=1:n,"midlon"=numeric(n),"midlat"=numeric(n))
  for (i in 1:n)
  {
    nodes$midlon[i] <- mean(cludata$lon[cludata$clust==i])
    nodes$midlat[i] <- mean(cludata$lat[cludata$clust==i])
  }

  nt <- matrix(rep(0,n*dtN),nr=n,nc=dtN)
  adj <- matrix(rep(0,n*n),nrow=n,ncol=n)
  Jijt <- array(rep(0,n*n*dtN),dim=c(n,n,dtN))
  ydgrz <- seq(0,365,len=(dtN+1))

  uid <- unique(cludata$idv)
  for (k in seq(along=uid))
  {
    stopk <- cludata[cludata$idv==uid[k],]
    arryd <- as.POSIXlt(stopk$time)$yday + as.POSIXlt(stopk$time)$hour/24 +
as.POSIXlt(stopk$time)$min/24/60 + as.POSIXlt(stopk$time)$sec/24/3600
    if (any(arryd>365)) arryd[arryd>365] <- 365
```

```
    sites <- numeric(dtN)

    for (i in seq(along=stopk[,1])[-1])
    {
      if (arryd[i-1]>arryd[i])
      {
        sites[which(ydgrz>=arryd[i-1] | ydgrz<arryd[i])] <- stopk$clust[i-
1]
      } else sites[which(ydgrz>=arryd[i-1] & ydgrz<arryd[i])] <-
stopk$clust[i-1]
    }
    if (arryd[length(arryd)]>arryd[1])
    {
      sites[which(ydgrz<arryd[1] | ydgrz>=arryd[length(arryd)])] <-
stopk$clust[length(arryd)]
    } else sites[which(ydgrz<arryd[1] & ydgrz>=arryd[length(arryd)])] <-
stopk$clust[length(arryd)]

    sites <- sites[1:dtN]

    for (t in 1:dtN) nt[sites[t],t] <- nt[sites[t],t] + 1

    for (j in seq(along=sites[-1])[-1])
    {
      if (sites[j] != sites[j-1])
      {
        adj[sites[j],sites[j-1]] <- adj[sites[j],sites[j-1]] + 1
        transit <- j-1
        Jijt[sites[j],sites[j-1],transit] <- Jijt[sites[j],sites[j-
1],transit] + 1
      }
    }
  }


  out <- which(rowSums(nt)==0)
  if (length(out)>0)
  {
    nt <- nt[-out,]
    print(c(Jijt[out,,],Jijt[,out,]))
    Jijt <- Jijt[-out,-out,]
    adj <- adj[-out,-out]
```

```
    print("Careful! Had to delete (an) unused node(s).")
  }

  return(list("nt"=nt,"adj"=adj,"Jijt"=Jijt))
}

##
circ.weight.mean <- function(x,w)
{
  my <- (1/sum(w)) * sum(w * sin(x))
  mx <- (1/sum(w)) * sum(w * cos(x))
  gM <- atan2 ( my, mx )
  if (gM < 0) gM <- gM + 2*pi
  return(gM)
}

circ.weight.sd <- function(xmean,xi,w)
{
  diff <- xi
  leng <- length(xi)
  for (i in 1:leng) diff[i] <- min( abs(xi[i]-xmean), abs( abs(xi[i]-
xmean)-2*pi))
  sd <- sqrt( (1/sum(w)) * sum(diff^2 * w))
  return(sd)
}

fitpij <- function(phi, h, s, t)
{
  f <- h * exp( ( cos( t-phi )-1 ) / (s^2) )
  return(f)
}

##
calc_fit_Rijt <- function(Jijt,nt)
{
  n <- dim(Jijt)[1]
  dtN <- dim(Jijt)[3]
  rijt <- Jijt

  timest <- 2*pi/dtN
  dtmid <- seq(timest/2,2*pi-timest/2,len=dtN)
```

```
  for (j in 1:dtN)
  {
    for (k in 1:n)
    {
      for (l in 1:n)
      {
        if (Jijt[k,l,j]>0) rijt[k,l,j] <- Jijt[k,l,j]/(nt[l,j]*timest)
      }
    }
  }

  phij <- hij <- sij <- matrix(rep(0,n*n),nc=n)
  for (k in 1:n)
  {
    for (j in 1:n)
    {
      posit <- which(rijt[k,j,]!=0 & j!=k)
      if (length(posit)>=1)
      {
        # (1) height
        hij[k,j] <- mean(rijt[k,j,posit])
        # (2) phase
        phij[k,j] <- circ.weight.mean(dtmid[posit],rijt[k,j,posit])
        # (3) width
        sij[k,j] <- circ.weight.sd
(xmean=phij[k,j],dtmid[posit],rijt[k,j,posit])
        if (sij[k,j]<0.01) sij[k,j] <- 3.5/365 *2*pi
      }
    }
  }

  rijfit <- array(rep(0,n*n*dtN),dim=c(n,n,dtN))
  for (k in 1:n)
  {
    for (j in 1:n)
    {
      posit <- which(rijt[k,j,]!=0 & j!=k)
      if (length(posit)>=1)
      {
        rijfit[k,j,] <- fitpij(phij[k,j],hij[k,j],sij[k,j],dtmid)
```

```
        #plot(dtmid,rijfit[k,j,],type="l",ylim=c(0,1))
        #points(dtmid,rijt[k,j,],type="h",lwd=2)
        #print(paste(k,j))
      }
    }
  }

  for (i in 1:n)
  {
    for (k in 1:dtN)
    {
      rijfit[i,i,k] <- -sum(rijfit[-i,i,k])
    }
  }
  return(list("rijfit"=rijfit,"phij"=phij,"hij"=hij,"sij"=sij))
}



calc_stable_Nit <- function(rijfit)
{
  require(expm)
  n <- dim(rijfit)[1]
  dtN <- dim(rijfit)[3]

  timest <- 2*pi/dtN
  dtmid <- seq(timest/2,2*pi-timest/2,len=dtN)

  Pijt <- array(rep(0,n*n*dtN),dim=c(n,n,dtN))
  for (k in 1:dtN) Pijt[,,k] <- expm(rijfit[,,k]*timest)

  Sijt <- array(rep(0,n*n*dtN),dim=c(n,n,dtN))
  for (ti in 1:dtN)
  {
    ix <- c(ti:dtN,1:(ti-1))[1:dtN]
    tmp <- Pijt[,,ix[1]]
    for (t in 2:dtN)
    {
      tmp <- Pijt[,,ix[t]] %*% tmp
    }
    Sijt[,,ti] <- tmp
```

```
  }

  x<-matrix(rep(0,n*dtN),nr=n)
  for (i in 1:dtN)
  {
    x[,i]<- abs(eigen(Sijt[,,i])$vectors[,1])
  }

  for (i in 1:dtN) x[,i] <- x[,i]/sum(x[,i])

  return(list("Nit"=x,"Pijt"=Pijt,"Sijt"=Sijt))
}

#########################################
#########################################
# run functions

#rm(list=ls())
setwd("./myfolder/")
options(digits.secs=0,stringsAsFactors=FALSE,max.print=99999)
Sys.setenv(tz="GMT")

spp <- "stork" # "stork" or "geese"
dtr <- read.csv(paste(spp,"fully_ad_daily_rastkoord_2018.csv",sep=""),
header=TRUE)

nw <- rast2NW(dtr,750,3)
counts <- count_pit_rijt(nw$cludata,dtN=48)
rates <- calc_fit_Rijt(counts$Jijt,counts$nt)
Nstab <- calc_stable_Nit(rates$rijfit)
```
